# Psychometric Properties of the Spanish Version of the Caregiver Burden Inventory

**DOI:** 10.3390/ijerph16020217

**Published:** 2019-01-14

**Authors:** Fernando L. Vázquez, Patricia Otero, Miguel A. Simón, Ana M. Bueno, Vanessa Blanco

**Affiliations:** 1 Department of Clinical Psychology and Psychobiology, University of Santiago de Compostela, 15782 Santiago de Compostela, Spain; 2 Department of Psychology, University of A Coruña, 15701 A Coruña, Spain; patricia.otero.otero@udc.es (P.O.); miguel.angel.simon@udc.es (M.A.S.); ana.bueno@udc.es (A.M.B.); 3 Department of Evolutionary and Educational Psychology, University of Santiago de Compostela, 15782 Santiago de Compostela, Spain; vanessa.blanco@usc.es

**Keywords:** Caregiver Burden Inventory, psychometric properties, internal consistency, factor analysis, burden, caregivers, Spanish

## Abstract

Although the Caregiver Burden Inventory (CBI) is the most widely used multidimensional burden instrument for assessing perceived burden of caregivers, there is no data on its psychometric properties in Spanish, nor on caregivers of dependent persons with various diseases. The objective of this study was to translate the CBI into Spanish and validate it in caregivers of dependent persons with various diseases. Trained evaluators administered the CBI and assessed emotional distress and probable mental disorder in 201 caregivers (87.1% women, mean age 56.2 years). The internal consistency of the CBI was 0.89 (0.74–0.83 among the subscales). There was a significant correlation of emotional distress with both the total burden and each subscale (*p* < 0.001 in all cases). A total score of 39 and scores of 16, 9, 8, 4, and 2 in burden per time dedicated to care, personal life burden, physical burden, social burden, and emotional burden were suitable cut-off points to discriminate caregivers with probable mental disorder (sensitivity = 63.0%–75.6%, specificity = 63.4%–74.4%). To achieve a greater goodness of fit, the model was re-specified, resulting in a shortened (15-item) instrument. The internal consistency reliability coefficients of the 15-item CBI were satisfactory (Cronbach *α* = 0.83; 0.77–0.86 among the subscales). Within the 15-item CBI, emotional distress was significantly correlated with the total burden, personal life burden, physical burden, social burden (*p* < 0.001 in all those cases), and emotional burden (*p* = 0.001). A total score of 25 and scores of 12, 5, 5, 3, and 1, respectively, in the subscales were identified as cut-off points to discriminate caregivers with probable mental disorder (sensitivity = 46.2%–70.6%, specificity = 43.9%–79.3%). Therefore, the 15-item CBI validly measured caregiver burden with better fit and more parsimoniously than the original CBI.

## 1. Introduction

Dependency has demonstrated rapid growth in recent years [1]. Although the availability of care and services for may differ significantly across countries [2], in the countries of the Organization for Economic Cooperation and Development more than one in 10 adults assumes the role of non-professional caregiver [3].

Nevertheless, caring for a dependent loved one usually extends over time and requires long hours of daily dedication (e.g., [4,5]), which can negatively impact the lives of caregivers. Most situations of care result in a decrease of free time and a deterioration of family relationships (80.2% of cases), damage to working life and financial stability (61.1% of cases), and a worsening of perceived health (55.6% of cases) [6]. In addition, often the social support that caregivers receive by people around them is limited [7]. Thus, caring for someone else becomes a source of stress that can negatively impact the caregiver and generate consequences defined as *burden of the caregiver*.

The conceptualization of caregiver burden has evolved over time. Initially it was defined as “any cost to the family” [8], where it was considered a global and one-dimensional construct. Then it was understood as a two-dimensional concept that encompasses the objective burden (i.e., activities and demands of care) and the subjective burden (i.e., attitudes and emotional reaction of the caregiver) [9]. Now caregiver burden is understood as a multidimensional construct that includes physical, psychological, emotional, social, and economic consequences [10,11,12]. This conceptualization of burden is in line with the stress model adapted for the caregiver population, which includes stressors directly and indirectly related to care [13].

Caregivers experiencing high burden are at greater risk of experiencing psychological distress [14], anxiety and depression [15,16] and report lower quality of life [17]. As a result, the quality of care they provide may be diminished, and in some cases, negligence and mistreatment of the dependent individual have been reported [18].

Therefore, the evaluation and detection of the burden is crucial. Among the currently available burden assessment instruments, the Caregiver Burden Inventory (CBI) [19] is the most widely used in the scientific literature that is consistent with the current concept of burden. The CBI is a multidimensional measure that evaluates different manifestations of caregiver burden, allowing the identification of the specific needs of each caregiver [20,21,22]. For its construction, 16 items were generated based on the experiences of 64 caregivers; subsequently, 8 items were added, selected from the review of the scientific literature. The final questionnaire consists of 24 items whose psychometric properties were analyzed in a sample of 171 caregivers of people with Alzheimer’s disease or cognitive impairment. The original English version has five factors: *time dependence burden, developmental burden, physical burden, social burden,* and *emotional burden*, whose internal consistencies were 0.85, 0.85, 0.86, 0.73, and 0.77, respectively.

The original English version of the CBI has subsequently been validated in Italian [16] and Chinese [23] with caregivers of elderly people with dementia, and in Portuguese with caregivers of elderly people [24]. However, despite the large number of non-professional caregivers in Spain (and other Spanish-speaking countries) [1,25] it has not been validated in Spanish, and it is unknown whether the original structure of the CBI fits to the data of Spanish samples and to caregivers of dependent persons with various diseases. In addition, although its subscales are positively correlated with anxiety and depression [15,16], it is unknown whether there is an adequate cut-off point to discriminate probable cases of mental disorder. The objective of this study was to translate the CBI into Spanish and validate it using confirmatory factor analysis, analyzing its psychometric properties in non-professional caregivers of dependent persons with various diseases.

## 2. Materials and Methods

### 2.1. Sample

A cross-sectional study was conducted. The sample was selected by simple random sampling from the official register of caregivers of the Ministry of Labor and Welfare of the Xunta de Galicia (Spain), a region of 29,434 km^2^ in northwest Spain with 2,732,347 inhabitants. For this, we signed an agreement with this institution to facilitate contact with caregivers and we followed the coming steps: (1) Make a list of all the non-professional caregivers (*n* = 18,410); (2) Assign a sequential number to each subject (1, 2, 3, …, 18,410); (3) Figure out the sample size (*n* = 210); (4) Use a random number generator to select the sample, using our sampling frame from Step 2 and our sample size from Step 3 (i.e., 210 random numbers between 1 and 18,410 were generated).

To participate in this study, the participants had to: (a) be a family caregiver of a person whose dependence was officially recognized, (b) live with the person cared for, and (c) provide informed consent. Exclusion criteria included: (a) presenting with any difficulty in communication (e.g., not being able to read or write) or any condition that could interfere with participation in the study (e.g., significant cognitive impairment, severe visual impairment), or (b) having received psychological or pharmacological treatment in the last two months.

The response rate was 95.7%. Of the 210 caregivers contacted to participate in the study, 9 refused participation, resulting in a final sample of 201 caregivers. Of the 201 participants, 87.1% were women with a mean age of 56.2 years (*SD* = 10.1), 79.6% had a partner, 64.2% had attended elementary school, 55.7% had a monthly family income between 1000 and 1999 euros and 43.8% took care of their father or mother. Of the people cared for, 55.7% were women with an average age of 71.6 years (*SD* = 21.5), and 54.2% had a physical disability. On average, the participants had been caring for their family member for 14.5 years (*SD* = 11.7) and 16.2 h per day (*SD* = 5.3). The average score of emotional distress was 4.1 (*SD* = 3.2), with 59.2% presenting a probable case of mental disorder (Table 1).

The study was conducted in accordance with the Declaration of Helsinki, and the protocol was approved by the Bioethics Committee of the University of Santiago de Compostela (Code number 07092016). All subjects gave their informed consent for inclusion before they participated in the study. Participation was voluntary, without economic compensation or any incentive. 

### 2.2. Instruments

The characteristics of the participants were evaluated via an ad hoc questionnaire including sociodemographic variables (gender, age, marital status, educational level, and monthly income) and care situation characteristics (relationship with the dependent, dependent gender, dependent age, disease of the dependent, time dedicated to care, and daily hours dedicated to care). Caregiver burden was assessed by the CBI [19], with an internal consistency for each subscale in the original version of 0.85, 0.85, 0.86, 0.73, 0.77. Emotional distress was assessed with the General Health Questionnaire (GHQ-12) [26], Spanish version of Rocha, Pérez, Rodríguez-Sanz, et al. [27], whose internal consistency of the Spanish version is 0.86 for people under 65 and 0.90 for people 65 and older. A cut-off point of 2/3 discriminates possible cases of mental disorder [28].

### 2.3. Procedure

The aim of linguistic validation is to obtain translations that are conceptually equivalent to the original, comparable across languages, and easily understood by the people to whom the translated instrument is administered [29]. To adapt the original English CBI version for Spanish caregivers, it was translated following the recommendations of Guillemin, Bombardier and Beaton [30], Hambleton and Zenisky [31] and the International Test Commission [32], including forward and backward translation [33]. We used independent forward and backwards translators who were experienced in translating psychological instruments and native speakers of the target languages. The English version was first translated into Spanish (including instructions, items and response options) by four Spanish native-speaker researchers. This draft of the Spanish version was then back-translated by an English native-speaker translator who had no previous exposure to the original English version of the CBI. Discrepancies between the meaning of the translation and that of the original version were reviewed and discussed by the translators until consensus was reached. Then, a committee of experts within the domains of clinical and developmental psychology with expertise in the caregiver population judged the translation. The translation-backtranslation process was repeated and the committee discussed with the translators until a new consensus was obtained on the semantic, idiomatic, experiential and conceptual equivalence between the Spanish version and the original English version. This pre-final version was presented to 10 caregivers that were not included in the study, to ensure the understanding of the questionnaire. No additional modifications were necessary, and this final version was used in the study.

Caregivers were contacted through letters and phone calls. The characteristics of the study were explained to them and they were invited to participate. To minimize dropouts, we followed the data collection strategies for cross-sectional studies [34], such as making the presentation of the study attractive to participants, treating the participants with kindness, affection and respect, and avoiding collecting information in an invasive way. Information about the characteristics of the participants, the situation of care, caregiver burden and emotional distress was collected via self-report in public centers close to the caregivers’ homes by three psychologists, who were previously trained. The evaluation was completed in approximately 40 min. 

### 2.4. Data Analysis

To analyze the differences in the total burden score and its subscales as a function of the sociodemographic characteristics and the care situation, Student’s *t*-tests, analysis of variance (ANOVA) or Pearson’s correlations were used.

To analyze the internal consistency of the CBI, we calculated the Cronbach α coefficient. We calculated the Pearson correlations between the items and between the score of each item and the total corrected score (i.e., the total score without considering said item). 

We applied the maximum likelihood method to perform a confirmatory factor analysis to verify the factorial structure of the questionnaire. The goodness of fit between the model and the observed data was verified by the following indices: (a) a significant χ^2^_M_ (generalized likelihood ratio), (b) Root Mean Square Error of Approximation (RMSEA) values ≤ 0.06, (c) Goodness of Fit Index (GFI) > 0.90, (d) Adjusted Goodness-of-fit Index (AGFI) > 0.90, (e) Comparative Fit Index (CFI) close to 0.95, (f) Normalized Fit Index (NFI) close to 0.95, (g) lower values of Expected Cross Validation Index (ECVI) [35,36]. In addition, the standardized factor loadings for each item on its respective factor is required to be ≥0.50 [37]. 

To examine the criterion validity of the CBI, we used the Pearson correlation of the CBI with emotional distress, the Student *t*-test for independent samples and a discriminant classification analysis with the probable cases of mental disorder. A Receiver’s Operating Characteristics (ROC) curve analysis was performed to determine the optimal discriminative cut-off point for determining probable cases of mental disorder. The indices of sensitivity, specificity, positive predictive value (PPV), and negative predictive value (NPV) were calculated. 

To achieve a greater goodness of fit, the model was re-specified and the analyses were repeated. Specifically, those items that contributed less to their corresponding factor were eliminated and the three items with the highest factor loading for each factor were selected [37,38,39]. Subsequently, the same analyses were repeated with the shortened version of the instrument as those that had been conducted with the original CBI. The analyses were performed with the statistical package SPSS for Windows (version 20.0, IBMCorp., Armonk, NY, USA) and SPSS_Amos Graphics (version 25, IBM Corp., Meadville, PA, USA).

## 3. Results

### 3.1. Original CBI

#### 3.1.1. Burden and Sample Characteristics

There was significant variation in the total caregiver burden depending on the illness of the person cared for, *F*(3, 196) = 7.194, *p* < 0.001, with significantly lower burden on caregivers of people with physical disabilities compared to those of people with cognitive impairment (*p* < 0.001). The total burden score correlated positively and significantly with the age of the person cared for (*r* = 0.152, *p* = 0.032) and the daily hours of care (*r* = 0.171, *p* = 0.015). 

When analyzing the subscales, there were significant differences in burden per time dedicated to care depending on the illness of the person cared for, *F*(3, 196) = 13.53, *p* < 0.001, with significantly lower burden in the caregivers of people with physical disabilities compared to those of people with intellectual disabilities (*p* = 0.008), mental disorders (*p* = 0.011), and cognitive impairment (*p* < 0.001). The burden per time dedicated to care correlated positively and significantly with the daily care hours (*r* = 0.261, *p* < 0.001). 

There were also differences in personal life burden depending on the illness of the person being cared for, *F*(3, 196) = 5.68, *p* = 0.001, with significantly higher burden on the caregivers of people with cognitive impairment than on those of people with intellectual (*p* = 0.026) or physical (*p* = 0.002) disabilities. The personal life burden correlated positively and significantly with the age of the person cared for (*r* = 0.226, *p* = 0.001), and the daily hours of care (*r* = 0.153, *p* = 0.030). 

Physical burden differed depending on the illness of the person cared for, *F*(3, 196) = 5.38, *p* = 0.001, being significantly lower in caregivers of people with physical disabilities than in those with mental disorders (*p* = 0.041) and cognitive impairments (*p* = 0.005). Finally, single caregivers had a higher social burden than partnered caregivers *t*(199) = 3.06, *p* = 0.003. 

#### 3.1.2. Reliability Analysis

The mean CBI score was 42.0 (*SD* = 15.9, range 6–93). The average scores for each subscale were: 16.1 (*SD* = 3.3) on burden per time dedicated to care, 9.0 (*SD* = 5.2) on personal life burden, 9.5 (*SD* = 5.4) on physical burden, 5.2 (*SD* = 4.5) social burden, and 2.2 (*SD* = 3.0) on emotional burden. Among the responses of the caregivers, 36.4% of the items were scored as 0; 12.6% scored as 1; 18.4% scored as 2; 13.1% scored as 3 and 19.5% scored as 4. The mean of the items ranged from 0.14 for item 21 to 3.68 for item 2 (Table 2). The corrected item-total correlation coefficients were all significant (*p* < 0.001) and spanned from 0.15 for item 4 to 0.76 for item 9. The mean of the inter-item correlation coefficient was 0.253, with a minimum of −0.13 and a maximum of 0.79. 

The total CBI showed an internal consistency of 0.89. The Cronbach’s α was 0.74 in burden per time dedicated to care, 0.83 in personal life burden, 0.78 in physical burden, 0.75 in social burden, and 0.78 in emotional burden. 

#### 3.1.3. Analysis of Validity

##### (1) Factorial structure

**Figure 1:**
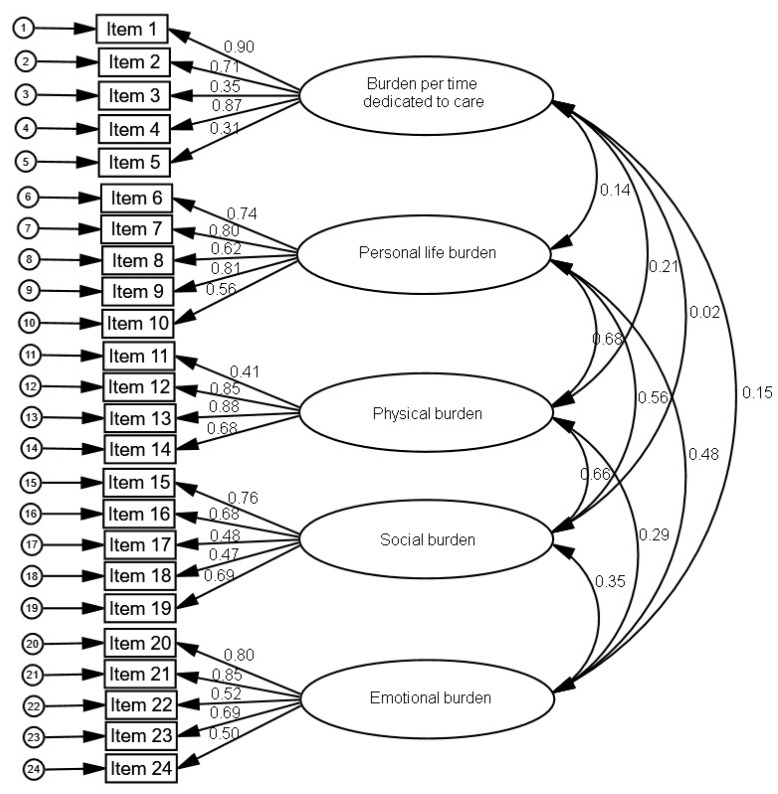
Results of the confirmatory factorial analysis for the original caregiver burden inventory (CBI).

In the confirmatory factor analysis, the adjustment indices were the following: χ^2^_M (242)_ = 704.07; *p* < 0.001, RMSEA = 0.098 (95% CI 0.089–0.106), GFI = 0.766, AGFI = 0.710, CFI = 0.789, NFI = 0.714 and ECVI = 4.100. Figure 1 shows the standardized loads and the covariances between factors. The items had significant factorial loads, ranging between 0.31 in item 5 and 0.90 in item 1. The covariances were significant between personal life and physical burden, personal life and social burden, physical and social burden. 

##### (2) Relationship between the original CBI and GHQ-12

The level of emotional distress was significantly positively correlated with the total burden score (*r* = 0.636, *p* < 0.001). In addition, caregivers with probable mental disorder presented higher burden scores than those without probable mental disorder, *t*(196) = −9.165, *p* < 0.001. 

**Figure 2:**
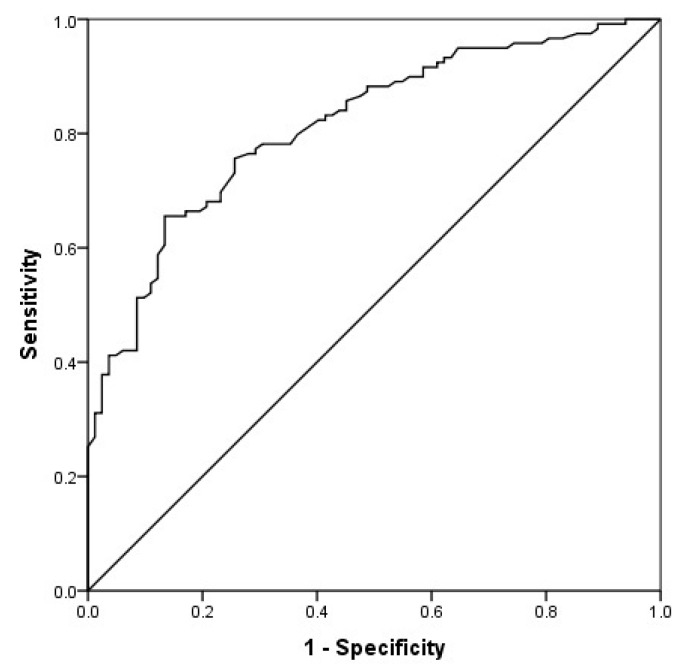
Receiver’s Operating Characteristics (ROC) Curve of the original CBI.

Using a discriminant classification analysis in the total CBI, Wilks’ lambda was 0.72, χ^2^(1, *n* = 201) = 64.80, *p* < 0.001. The canonical correlation, which measures association between discriminant scores and group membership, was 0.53. This analysis correctly classified 72.6% of the cases (Table 3). The area under the ROC curve was 0.81 (95% CI 0.75–0.87; Figure 2). For the cut-off point of 39, the test showed a sensitivity of 75.6%, specificity of 74.4%, PPV of 81.1%, and NPV of 67.8% (Table 4). 

Regarding subscales, there was a positive correlation between emotional distress and burden per time dedicated to care (*r* = 0.244, *p* < 0.001), personal life burden (*r* = 0.512, *p* < 0.001), physical burden (*r* = 0.583, *p* < 0.001), social burden (*r* = 0.590, *p* < 0.001) and emotional burden (*r* = 0.306, *p* < 0.001). Caregivers with probable cases of mental disorder, compared to those without it, had higher burden per time dedicated to care, *t*(199) = −3.99, *p* < 0.001, personal life burden, *t*(199) = −6.76, *p* < 0.001, physical burden, *t*(199) = −7.77, *p* < 0.001, social burden, *t*(197) = −7.33, *p* < 0.001, and emotional burden, *t*(193) = −3.65, *p* < 0.001. 

Wilks’ lambda ranged between 0.77 in physical burden and 0.94 in emotional burden. The canonical correlation was from 0.24 in emotional burden to 0.48 in physical burden. The percentages of correctly classified cases ranged between 58.7% and 71.6% (Table 3). The area under the ROC curve was 0.68 (95% CI 0.61–0.74) for burden per time dedicated to care, 0.76 (95% CI 0.69–0.81) for personal life burden, 0.78 (95% CI 0.72–0.84) for physical burden, 0.77 (95% CI 0.71–0.83) for social burden and 0.67 (95% CI 0.60–0.73) for emotional burden. The cutoff points of 16, 9, 8, 4, and 2 in the respective subscales showed a sensitivity between 63.0% and 74.0%, specificity between 63.4% and 72.0%, PPV between 71.7% and 78.9% and NPV between 54.7% and 64.1 % (Table 4). 

### 3.2. Shortened CBI

Because the 24-item Spanish version of the CBI did not fit strictly to the data, the model was re-specified. The items eliminated from the original CBI were the following: items 3 and 5 (belonging to the subscale *burden per time dedicated to care*), items 9 and 10 (belonging to the subscale *personal life burden*), item 11 (belonging to the subscale *physical burden*), items 17 and 18 (belonging to the subscale *social burden*), and items 22 and 24 (belonging to the subscale *emotional burden*). The elimination of these items resulted in a 15-item version of the CBI.

#### 3.2.1. Burden and Sample Characteristics

In the 15-item CBI, we found significant differences in the total caregiver burden depending on the illness of the person cared for, *F*(3, 196) = 5.312, *p* = 0.002, with significantly lower burden on caregivers of people with physical disabilities compared to those of people with cognitive impairment (*p* = 0.002). The total burden score correlated positively and significantly with the age of the person cared for (*r* = 0.176, *p* = 0.012) and the daily hours of care (*r* = 0.142, *p* = 0.045).

Regarding the subscales, the burden per time dedicated to care correlated positively and significantly with the daily care hours (*r* = 0.235, *p* = 0.001). There were differences in personal life burden depending on the illness of the person being cared for, *F*(3, 196) = 5.12, *p* = 0.002, with significantly higher burden on the caregivers of people with cognitive impairment than on those of people with intellectual (*p* = 0.031) or physical (*p* = 0.003) disabilities. The personal life burden also correlated positively and significantly with the age of the person cared for (*r* = 0.290, *p* < 0.001). Single caregivers had a higher social burden than partnered caregivers, *t*(199) = 3.61, *p* < 0.001. Finally, female caregivers had a higher emotional burden compared to male caregivers, *t*(71) = −2.24, *p* = 0.028.

#### 3.2.2. Reliability Analysis

The mean score of the 15-item CBI was 25.6 (*SD* = 9.31, range 5–57). The average scores for each subscale were: 11.0 (*SD* = 1.8) on burden per time dedicated to care, 4.9 (*SD* = 3.3) on personal life burden, 5.5 (*SD* = 3.5) on physical burden, 3.3 (*SD* = 3.4) on social burden, and 0.9 (*SD* = 1.8) on emotional burden. Among the responses of the caregivers, 37.5% of the items were scored as 0; 12.0% as 1; 15.6% as 2; 12.4% as 3 and 22.5% as 4. The mean of the items ranged from 0.14 for item 14 to 3.68 for item 2 (Table 5). The corrected item-total correlation coefficients were all significant (*p* < 0.001) and ranged from 0.17 for item 3 to 0.67 for item 8. The mean of the inter-item correlation coefficient was 0.243, with a minimum of −0.05 and a maximum of 0.79. 

The 15-item CBI had a total internal consistency of 0.83. The Cronbach’s α was 0.86 in burden per time dedicated to care, 0.77 in personal life burden, 0.84 in physical burden, 0.78 in social burden, and 0.79 in emotional burden.

#### 3.2.3. Analysis of Validity

##### (1) Factorial structure

**Figure 3:**
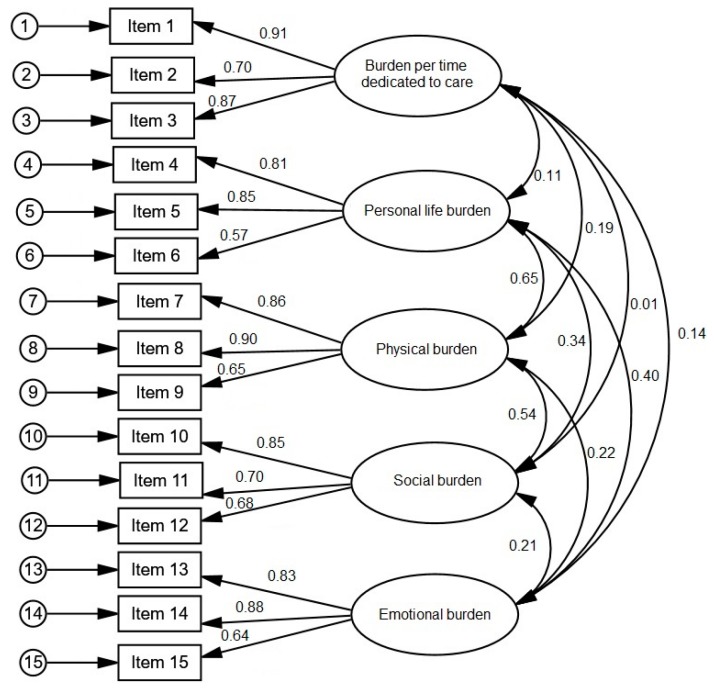
Results of the confirmatory factorial analysis for the 15-item CBI.

The confirmatory analysis of the 15-item CBI revealed the following fit indexes: χ^2^_M (242)_ = 138.36; *p* < 0.001, RMSEA = 0.060 (95% CI 0.043–0.077), GFI = 0.919, AGFI = 0.878, CFI = 0.957, NFI = 0.906, and ECVI = 1.092. The items had significant factorial loads, ranging between 0.57 in item 6 to 0.91 in item 1 (Figure 3). The covariances were significant between personal life and physical burden, personal life, and emotional burden, and physical and social burden.

##### (2) Relationship between the original CBI and GHQ-12

Emotional distress was significantly positively correlated with the total burden score of the 15-item CBI (*r* = 0.584, *p* < 0.001). Furthermore, caregivers with probable mental disorder presented with higher burden than those without probable mental disorder, *t*(196) = −7.499, *p* < 0.001. 

**Figure 4:**
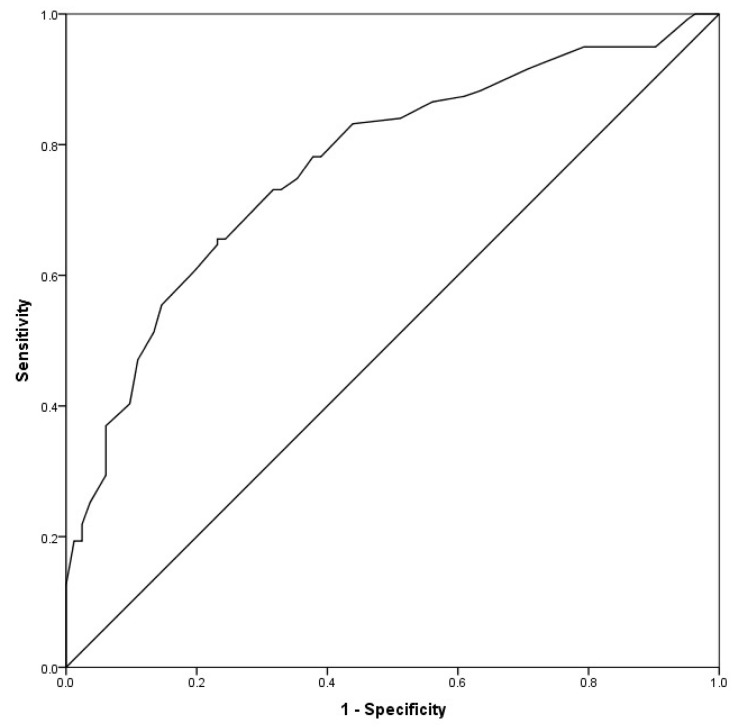
ROC Curve of the 15-item CBI.

The discriminant classification analysis in the total 15-item CBI, had a Wilks’ lambda of 0.80, χ^2^ (1, *n* = 201) = 45.56, *p* < 0.001 and a canonical correlation of 0.45. This analysis correctly classified 70.6% of cases (Table 6). The area under the ROC curve was 0.77 (95% CI 0.70–0.83); Figure 4). The cut-off point of 25 showed a sensitivity of 70.6%, specificity of 70.7%, PPV of 77.8%, and NPV of 62.4% (Table 7). 

Regarding subscales, there was a non-significant positive correlation between emotional distress and burden per time dedicated to care (*r* = 0.063, *p* = 0.371), but significant positive correlations between emotional distress and personal life burden (*r* = 0.443, *p* < 0.001), physical burden (*r* = 0.524, *p* < 0.001), social burden (*r* = 0.461, *p* < 0.001) and emotional burden (*r* = 0.238, *p* = 0.001). There was no difference between caregivers with or without probable cases of mental disorder in burden per time dedicated to care, *t*(199) = −1.13, *p* = 0.261. However, caregivers with a probable case of mental disorder, compared to those without it, had higher personal life burden, *t*(199) = −5.50, *p* < 0.001, physical burden, *t*(199) = −6.28, *p* < 0.001, social burden, *t*(194) = −5.10, *p* < 0.001, and emotional burden, *t*(192) = −2.59, *p* = 0.01. 

Wilks’ lambda ranged between 0.84 in physical burden and 0.99 in burden per time dedicated to care (which was non-significant). The canonical correlation was from 0.08 in burden per time dedicated to care to 0.41 in physical burden. The percentages of correctly classified cases ranged between 57.2% in the non-significant burden per time dedicated to care and 68.7% in physical burden (Table 6). The area under the ROC curve was 0.56 (95% CI 0.49–0.63) for burden per time dedicated to care, 0.71 (95% CI 0.65–0.78) for personal life burden, 0.74 (95% CI 0.67–0.80) for physical burden, 0.71 (95% CI 0.64–0.77) for social burden and 0.63 (95% CI 0.56–0.70) for emotional burden. The cutoff points of 12, 5, 5, 3 and 1 in the respective subscales showed a sensitivity between 46.2% and 70.6%, specificity between 43.9% and 79.3%, PPV between 61.3% and 76.4% and NPV between 47.1% and 60.7% (Table 7). 

## 4. Discussion

In this study, we translated the CBI to Spanish and examined the psychometric properties by administering it to a sample of caregivers of dependent persons with various diseases. In both, the original and a shortened version (15-item) of the CBI we found that the total burden was significantly lower in the caregivers of people with physical disabilities compared to people with cognitive impairments. In addition, total burden was positively correlated to the age of the person being cared for and the number of daily hours of care. It is possible that caring for people with cognitive impairment is more limiting due to their disruptive behaviors and the greater need for supervision [40], which can be accentuated by an advanced age and the amount of time dedicated to care. 

The internal consistency of the CBI was satisfactory (total Cronbach α = 0.89 in the original CBI and 0.83 in the 15-item CBI; between 0.74 and 0.83 in the five subscales of the CBI and slightly higher values between 0.77 and 0.86 in the subscales of the 15-item CBI). Because all the values were greater than 0.70, both the original and shortened versions of the CBI have an acceptable reliability [41]. Furthermore, these results are similar to those of the original instrument, whose values ranged between 0.73 and 0.86 [19], and are consistent with those reported in the Chinese, Italian and Portuguese versions of the instrument [16,23,24]. 

The results of the confirmatory analysis with the CBI found were not entirely satisfactory. Therefore, the model was re-specified, eliminating those items that contributed less to the corresponding factors and retaining the three items with the highest load for each factor [37,38,39]. Scientific literature recommends consistently a minimum of three items loading significantly on each factor in multidimensional scales [39,42]. A possible explication of the unsatisfactory goodness of fit could be that those removed items with lower factorial loadings are unrepresentative of the sample of our study due to some sociodemographic and cultural singularities of the same. Thus, items 3 and 5 (“I have to watch my care receiver constantly”, “I do not have a minute’s break from my caregiving chores”) are relevant for dementia caregivers (like in the original English version of the CBI), but can be not applicable to caregivers of people with other conditions like the 54.7% of caregivers of people with physical disability of our sample. Items 9, 10, 11, 17, 22, 24 (“I feel emotionally drained due to caring for my care receiver”, “I expected that things would be different at this point in my life”, “I am not getting enough sleep”, I have had problems with my marriage”, “I resent my care receiver” and “I feel angry about my interactions with my care receiver”, respectively) could fit less into Spanish culture in which the support and responsibility of families towards their dependent members has a long tradition [6,25]. Lastly, item 18 (“I do not do as good a job at work as I used to”) could not be representative for most of caregivers in Spain, because 73.1% of the caregivers do not have a job [6]. The resulting 15-item CBI revealed an acceptable, although moderate fit, for the five-factor model. In addition, at least half of the retained items in each factor had loadings ≥0.60, which support factor stability of this shortened version [43]. Overall, the Spanish version of the CBI was consistent with the five-factor structure of the original instrument [19], and also consistent with the Chinese [23] and Portuguese [24] versions. The covariances between factors indicated that they were not redundant, reflecting a multidimensional instrument, which is consistent with the multidimensional definition of caregiver burden [10,11,12].

Additionally, we found that a higher level of total burden in both the original and the shortened version of the CBI and all of their subscales except burden per time dedicated to care of the shortened version were associated with greater emotional distress. Further, caregivers with a probable mental disorder case had significantly higher scores than those who did not have a probable case of mental disorder on all subscales except burden per time dedicated to care of the shortened version. These results indicate that the CBI presents more concurrent validity and specificity to reflect the repercussions of care on the welfare of the caregiver in the complete version, which was consistent with previous research [16]. Finally, in the original CBI the cut-off points of 39 in the total score and of 16, 9, 8, 4, and 2 in the subscales (time dedicated to care, personal life burden, physical, social, and emotional burden) were adequate to discriminate between caregivers with and without probable mental disorders. Instead, the 15-item CBI showed scarce discrimination capacity: the cut-off points presented low sensitivity (42.6%) in emotional burden and low specificity (43.9%) in burden per time dedicated to care, with the consequent risk of false positives and false negatives.

### 4.1. Implications

This study has important implications for research, society and policymakers. It suggests that the burden borne by caregivers can be high enough to justify a referral to professionals for proper evaluation. The results show that the reliability and validity of the Spanish version of the CBI were generally supported, consistent with the original English version of the instrument. In addition, a shortened, 15-item version of the CBI that fit better with the cultural context and sociodemographic characteristics of the Spanish non-professional caregivers of dependent persons with various diseases validly measured caregiver burden. Given the reluctance of caregivers to complete long questionnaires due to their lack of available time, this 15-item version provide a more parsimonious instrument which reliably included all relevant dimensions. The 15-item version may improve efficiency of administration, making it an attractive choice for researchers and clinicians. However, future research is needed to replicate these results for the shortened version. Furthermore, it provides a cut-off point that discriminates caregivers with and without probable cases of mental disorder, although the results of this study suggest that the cut-off points of the 15-item CBI should be used cautiously. Future studies could analyze new cut-off points applicable to other specific mental health problems. 

The administration of this instrument would provide detailed information on the multidimensional manifestations of burden, facilitating the identification of different profiles of caregivers’ burden. Policymakers could use this instrument to understand the specific needs of the caregiver population. In addition, the instrument would also identify caregivers with excessive burden, thus allowing the study of protective and risk factors. Furthermore, this instrument is useful for researchers and clinicians to distinguish potential areas of intervention for caregivers, which would allow the development of interventions tailored to the needs of specific caregivers and the evaluation of caregiver burden post-intervention. Given the high prevalence of caregivers in Spain (and other Spanish-speaking countries) [1,25], the Spanish version of the CBI benefit a large number of caregivers in the present and the future.

### 4.2. Limitations

The current study is not without limitations. We used the GHQ-12 to assess probable cases of mental disorder instead of a diagnostic interview. Although this is a commonly used instrument, it does not establish clinical diagnoses. In addition, the self-reported nature of the instruments used could exacerbate the common variance and artificially increase the correlations between variables [44]. Also, reliance on self-reported instruments may introduce response bias due to social desirability, acquiescence and common scale anchors. To reduce them, we followed recommendations by Podsakoff, MacKenzie, Lee et al. [45], including protecting respondent anonymity, assuring participants that there were no right or wrong answers and asking them to answer questions as honestly as possible. Self-reported instruments for predictor and criterion measures had different scale endpoints and formats, the predictor measurement (GHQ-12) had sound psychometric properties [46], and bipolar scale values were avoided. Another limitation is that the size of the sample was insufficient to perform a yardstick of the instrument to address different demographic segments. Finally, the fact that the sample of caregivers was from one of regions of Spain (Galicia) limits the external validity, although the data available in other regions of our country have a similar demographic and clinical profile [25]. 

## 5. Conclusions

In conclusion, the results of this study provided evidence of a five-factor structure and good reliability of the five subscales in the Spanish CBI. The 15-item abbreviated version of the CBI has acceptable psychometric properties to assess the burden on the population of Spanish non-professional caregivers of dependent persons with various diseases, though it has a low capacity of discrimination between caregivers with and without probable mental health disorder.

## Figures and Tables

**Table 1:** Sociodemographic, care and clinical characteristics.

Variables	*n* = 201	%
Gender		
Male	26	12.9
Female	175	87.1
Age		
*M (SD)*	56.2 (10.1)	
Marital status		
Single	41	20.4
Partnered	160	79.6
Educational level		
Literate	30	14.9
Elementary school	129	64.2
High school–college	42	20.9
Monthly income		
<999 euros	71	35.3
Between 1000 and 1999 euros	112	55.7
>2000 euros	18	9.0
Relationship with the dependent		
Parent	88	43.8
Spouse	20	10.0
Daughter/son	42	20.9
Other relatives	45	22.4
Not related	6	3.0
Dependent gender		
Male	89	44.3
Female	112	55.7
Dependent age		
*M (SD)*	71.6 (21.5)	
Disease of the dependent		
Intellectual disability	19	9.5
Mental disorder	16	8.0
Physical disability	110	54.7
Cognitive impairment	56	27.9
Time dedicated to care (years)		
*M (SD)*	14.5 (11.7)	
Daily hours dedicated to care		
*M (SD)*	16.2 (5.3)	
Emotional distress		
*M (SD)*	4.1 (3.2)	
Probable case of mental disorder		
Yes	119	59.2
No	82	40.8

**Table 2:** Means, standard deviations, score frequency and corrected item-total correlation (*r*^tot^) for each item of the original Caregiver Burden Inventory (CBI).

Items	*M*	*SD*	Score Frequency (%)					*r* ^tot^
			0	1	2	3	4	
**Carga por tiempo de dedicación (Time-dependence burden)**								
1. La persona que cuido necesita mi ayuda para llevar a cabo muchas tareas diarias (My care receiver needs my help to perform many daily tasks)	3.66	0.66	0.5	1.0	4.5	20.4	73.6	0.18
2. La persona que cuido depende de mi (My care receiver is dependent on me)	3.68	0.60	0.0	0.5	5.5	19.4	74.6	0.29
3. Tengo que vigilar constantemente a la persona que cuido (I have to watch my care receiver constantly)	2.81	1.28	9.0	6.5	20.4	23.4	40.7	0.46
4. Tengo que ayudar a la persona que cuido en muchas actividades básicas (I have to help my care receiver with many basic functions)	3.64	0.72	1.0	1.0	5.5	18.4	74.1	0.15
5. No tengo ni un minuto de descanso de mis labores de cuidado (I do not have a minute’s break from my caregiving chores)	2.31	1.21	10.0	12.4	33.8	23.9	19.9	0.47
**Carga en la vida personal (Developmental burden)**								
6. Siento que estoy desperdiciando mi vida (I feel that I am missing out on life)	1.41	1.31	35.3	18.4	25.4	12.4	8.5	0.56
7. Desearía poder escapar de esta situación (I wish I could escape from this situation)	1.34	1.24	35.8	17.9	29.4	10.4	6.5	0.63
8. Mi vida social se ha deteriorado (My social life has suffered)	2.10	1.45	22.9	9.0	25.8	19.4	22.9	0.57
9. Me siento emocionalmente agotado debido a la atención dedicada a la persona que cuido (I feel emotionally drained due to caring for my care receiver)	1.88	1.28	19.9	15.9	34.4	16.9	12.9	0.76
10. Esperaba que las cosas fueran diferentes en este momento de mi vida (I expected that things would be different at this point in my life)	2.26	1.45	20.9	6.5	24.4	22.9	25.3	0.44
**Carga física (Physical burden)**								
11. No consigo dormir lo suficiente (I am not getting enough sleep)	2.10	1.45	23.4	8.0	25.8	20.9	21.9	0.47
12. Mi salud se ha deteriorado (My health has suffered)	1.84	1.39	25.4	15.9	23.4	20.9	14.4	0.62
13. Ser cuidador me ha hecho enfermar físicamente (Caregiving has made me physically sick)	1.47	1.38	36.3	17.9	17.4	19.4	9.0	0.67
14 Estoy cansado físicamente (I am physically tired)	2.18	1.28	14.4	11.4	35.9	18.9	19.4	0.60
**Carga social (Social burden)**								
15. No me llevo tan bien como antes con otros miembros de mi familia (I do not get along with other family members as well as I used to)	0.97	1.31	53.7	19.4	12.4	5.0	9.5	0.50
16. Mis esfuerzos como cuidador no son apreciados por otros miembros de mi familia (My caregiving efforts are not appreciated by others in my family)	1.32	1.40	41.3	18.9	17.9	10.0	11.9	0.44
17. He tenido problemas en mi matrimonio (I have had problems with my marriage)	0.76	1.12	60.2	17.4	11.4	8.0	3.0	0.49
18. No hago tan bien mis tareas en el trabajo como solía hacerlo (I do not do as good a job at work as I used to)	1.09	1.13	41.2	22.9	23.9	9.0	3.0	0.52
19. Me siento resentido con otros familiares que podrían ayudar pero no lo hacen (I feel resentful of other relatives who could but do not help)	1.03	1.34	52.2	18.9	11.9	7.5	9.5	0.48
**Carga emocional (Emotional burden)**								
20. Me avergüenza el comportamiento de la persona que cuido (I feel embarrassed over my care receiver’s behavior)	0.31	0.72	80.1	12.4	5.0	1.5	1.0	0.41
21. Me siento avergonzado de la persona que cuido (I feel ashamed of my care receiver)	0.14	0.55	92.0	5.0	1.5	0.5	1.0	0.33
22. Estoy resentido con la persona que cuido (I resent my care receiver)	0.32	0.74	80.6	10.9	6.0	1.5	1.0	0.43
23. Me siento incómodo cuando tengo amigos de visita (I feel uncomfortable when I have friends over)	0.49	0.88	71.2	13.9	11.4	2.0	1.5	0.48
24. Me enfadan mis interacciones con la persona a la que cuido (I feel angry about my interactions with my care receiver)	0.98	1.07	45.7	20.4	27.4	3.5	3.0	0.45
Total Cronbach’s α								0.89
Burden per time dedicated to care—Cronbach’s α								0.74
Personal life burden—Cronbach’s α								0.83
Physical burden—Cronbach’s α								0.78
Social burden—Cronbach’s α								0.75
Emotional burden—Cronbach’s α								0.78
Mean inter-item correlation coefficient								0.253

**Table 3:** Discriminant analysis and percentage of cases correctly classified of the original CBI.

Total/Subscales	Wilks’ Lambda (χ^2^)	Canonical Correlation	Probable Cases of Mental Disorder	No Cases of Mental Disorder	Total
Total burden	0.72 (64.80) **	0.53	69.7	76.8	72.6
Burden per time dedicated to care	0.93 (15.25) **	0.27	70.6	50.0	62.2
Personal life burden	0.81 (41.02) **	0.43	72.3	70.7	71.6
Physical burden	0.77 (51.39) **	0.48	68.1	73.2	70.1
Social burden	0.80 (43.42) **	0.44	66.4	73.2	69.2
Emotional burden	0.94 (11.97) *	0.24	43.7	80.5	58.7

Note: ** *p* < 0.001; * *p* = 0.001.

**Table 4:** Predictive ability for the cut-off points of the original CBI.

Total/Subscales	Cut-Off Point	Sensitivity	Specificity	Positive Predictive Value	Negative Predictive Value
Total burden	39	75.6	74.4	81.1	67.8
Burden per time dedicated to care	16	70.6	63.4	71.7	54.7
Personal life burden	9	72.3	72.0	78.9	64.1
Physical burden	8	72.3	65.9	75.4	62.1
Social burden	4	74.0	65.9	75.9	63.5
Emotional burden	2	63.0	65.9	72.8	55.1

**Table 5:** Means, standard deviations, score frequency and corrected item-total correlation (*r*^tot^) for each item of the 15-item CBI.

Items	*M*	*SD*	Score Frequency (%)					*r* ^tot^
			0	1	2	3	4	
**Carga por tiempo de dedicación (Time-dependence burden)**								
1. La persona que cuido necesita mi ayuda para llevar a cabo muchas tareas diarias (My care receiver needs my help to perform many daily tasks)	3.66	0.66	0.5	1.0	4.5	20.4	73.6	0.21
2. La persona que cuido depende de mi (My care receiver is dependent on me)	3.68	0.60	0.0	0.5	5.5	19.4	74.6	0.28
3. Tengo que ayudar a la persona que cuido en muchas actividades básicas (I have to help my care receiver with many basic functions)	3.64	0.72	1.0	1.0	5.5	18.4	74.1	0.17
**Carga en la vida personal (Developmental burden)**								
4. Siento que estoy desperdiciando mi vida (I feel that I am missing out on life)	1.41	1.31	35.3	18.4	25.4	12.4	8.5	0.51
5. Desearía poder escapar de esta situación (I wish I could escape from this situation)	1.34	1.24	35.8	17.9	29.4	10.4	6.5	0.56
6. Mi vida social se ha deteriorado (My social life has suffered)	2.10	1.45	22.9	9.0	25.8	19.4	22.9	0.53
**Carga física (Physical burden)**								
7. Mi salud se ha deteriorado (My health has suffered)	1.84	1.39	25.4	15.9	23.4	20.9	14.4	0.63
8. Ser cuidador me ha hecho enfermar físicamente (Caregiving has made me physically sick)	1.47	1.38	36.3	17.9	17.4	19.4	9.0	0.67
9. Estoy cansado físicamente (I am physically tired)	2.18	1.28	14.4	11.4	35.9	18.9	19.4	0.57
**Carga social (Social burden)**								
10. No me llevo tan bien como antes con otros miembros de mi familia (I do not get along with other family members as well as I used to)	0.97	1.31	53.7	19.4	12.4	5.0	9.5	0.52
11. Mis esfuerzos como cuidador no son apreciados por otros miembros de mi familia (My caregiving efforts are not appreciated by others in my family)	1.32	1.40	41.3	18.9	17.9	10.0	11.9	0.45
12. Me siento resentido con otros familiares que podrían ayudar pero no lo hacen (I feel resentful of other relatives who could but do not help)	1.03	1.34	52.2	18.9	11.9	7.5	9.5	0.47
**Carga emocional (Emotional burden)**								
13. Me avergüenza el comportamiento de la persona que cuido (I feel embarrassed over my care receiver’s behavior)	0.31	0.72	80.1	12.4	5.0	1.5	1.0	0.38
14. Me siento avergonzado de la persona que cuido (I feel ashamed of my care receiver)	0.14	0.55	92.0	5.0	1.5	0.5	1.0	0.32
15. Me siento incómodo cuando tengo amigos de visita (I feel uncomfortable when I have friends over)	0.49	0.88	71.2	13.9	11.4	2.0	1.5	0.43
Total Cronbach’s α								0.83
Burden per time dedicated to care—Cronbach’s α								0.86
Personal life burden—Cronbach’s α								0.77
Physical burden—Cronbach’s α								0.84
Social burden—Cronbach’s α								0.78
Emotional burden—Cronbach’s α								0.79
Mean inter-item correlation coefficient								0.243

**Table 6:** Discriminant analysis and percentage of cases correctly classified of the 15-item CBI.

Total/Subscales	Wilks’ Lambda (χ^2^)	Canonical Correlation	Probable Cases of Mental Disorder	No Cases of Mental Disorder	Total
Total burden	0.80 (45.56) **	0.45	70.6	70.7	70.6
Burden per time dedicated to care	0.99 (1.27)	0.08	77.3	28.0	57.2
Personal life burden	0.87 (28.07) **	0.36	68.1	62.2	65.7
Physical burden	0.84 (35.87) **	0.41	66.4	72.0	68.7
Social burden	0.89 (22.71) **	0.33	54.6	76.8	63.7
Emotional burden	0.97 (6.15) *	0.18	46.2	80.5	60.2

Note: ** *p* < 0.001; * *p* = 0.001.

**Table 7:** Predictive ability for the cut-off points of the 15-item CBI.

Total/Subscales	Cut-Off Point	Sensitivity	Specificity	Positive Predictive Value	Negative Predictive Value
Total burden	25	70.6	70.7	77.8	62.4
Burden per time dedicated to care	12	68.1	43.9	61.3	47.1
Personal life burden	5	68.1	63.4	73.0	57.8
Physical burden	5	70.6	65.9	75.0	60.7
Social burden	3	64.7	68.3	74.8	57.1
Emotional burden	1	46.2	79.3	76.4	50.4
